# Revisiting the Chemistry of Vinylpyrazoles: Properties, Synthesis, and Reactivity

**DOI:** 10.3390/molecules27113493

**Published:** 2022-05-29

**Authors:** Vera L. M. Silva, Artur M. S. Silva

**Affiliations:** LAQV-REQUIMTE, Department of Chemistry, University of Aveiro, 3810-193 Aveiro, Portugal; artur.silva@ua.pt

**Keywords:** pyrazoles, vinylpyrazoles, biological activity, synthesis, reactivity

## Abstract

Vinylpyrazoles, also known as pyrazolyl olefins, are interesting motifs in organic chemistry but have been overlooked. This review describes the properties and synthetic routes of vinylpyrazoles and highlights their versatility as building blocks for the construction of more complex organic molecules. Concerning the reactivity of vinylpyrazoles, the topics surveyed herein include their use in cycloaddition reactions, free-radical polymerizations, halogenation and hydrohalogenation reactions, and more recently in transition-metal-catalyzed reactions, among other transformations. The current state of the art about vinylpyrazoles is presented with an eye to future developments regarding the chemistry of these interesting compounds. Styrylpyrazoles were not considered in this review, as they were the subject of a previous review article published in 2020.

## 1. Introduction

**Figure 1:**
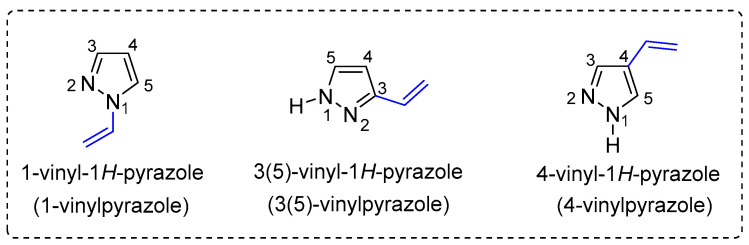
Structures of 1-vinylpyrazole, 3(5)-vinylpyrazoles, and 4-vinylpyrazole. For sake of simplicity, throughout the manuscript, the nomenclature 1-vinylpyrazole is adopted instead of 1-vinyl-1*H*-pyrazole.

Pyrazoles have attracted increased attention in recent years owing to their widespread applications in medicine [1,2,3,4,5,6,7,8,9,10,11,12,13,14,15], agriculture, [16] materials science [17,18,19,20], and catalysis [21,22]. In this review, the chemistry of a particular kind of pyrazoles, the vinylpyrazoles, is revisited. Vinylpyrazoles (vinyl-1*H*-pyrazoles), also known as pyrazolyl olefins, are characterized by the presence of a vinyl group linked at one of the pyrazoles’ ring positions (N-1, C-3, C-4, C-5) (Figure 1). There are few examples of vinylpyrazoles despite their interesting properties. These compounds are interesting building blocks for the synthesis of more complex pyrazoles endowed with biological activities and for other advanced structures, such as indazoles, among others. For example, some 5-vinylpyrazoles were found to be potent DNA gyrase inhibitors with antibacterial activity against Gram-(+) bacteria [23], while others have been used as additives in the production of rubbers [24,25,26]. Furthermore, these compounds present interesting physicochemical properties, such as tautomerism and isomerism, owing to the presence of the vinyl group. Consequently, several spectroscopic studies on vinylpyrazoles have been reported [27,28,29,30,31,32,33,34,35]. For example, Skvortsova and coworkers performed several ^1^H- and ^13^C-NMR spectral analyses for evaluation of electronic and steric effects in 1-vinylpyrazoles [28]. They showed that the substituents on the pyrazole ring had an effect on the conformation of the vinyl group; 5-methyl-1-vinylpyrazoles had predominantly S-*cis*-N^2^ orientation, while 1-vinylpyrazoles without substituents at C-5 were a mixture of conformers. These observations were confirmed six years later by Vashchenko and Afonin [35]. Despite their interesting structural features and chemistry, vinylpyrazoles have been overlooked, and their reactivity has not been much explored. However, the discovery of several tools in modern organic synthesis in the last decades, mainly related to metathesis, transition-metal-catalyzed and C-H activation reactions, and visible light-driven and green organometallic transformations, among others, may open novel opportunities towards the investigation of the vinylpyrazoles reactivity and versatility in organic synthesis. This review intends to show the current state of the art with an eye to future perspectives concerning the chemistry and reactivity studies of vinylpyrazoles.

## 2. Synthesis of Vinylpyrazoles

**Scheme 1:**
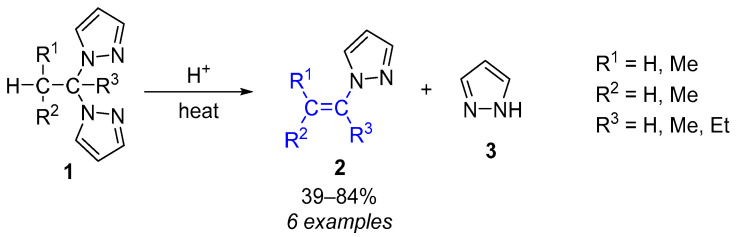
Synthesis of 1-vinylpyrazoles **2** from geminal bis(1-pyrazolyl)alkanes **1** [38].

One of the first methods described in the literature for the synthesis of 1-vinylpyrazoles, namely 3,5-dimethyl-1-vinylpyrazole and 3-methyl-5-phenyl-1-vinylpyrazole, was the reaction of acetylene with 3,5-dimethylpyrazole and 3-methyl-5-phenylpyrazole using high pressure [36]. The compounds 1-vinylpyrazole and 3,5-dimethyl-1-vinylpyrazole were also prepared by dehydration of the corresponding alcohols, 1-(β-hydroxyethyl)pyrazole and 3,5-dimethyl-1-(β-hydroxyethyl)pyrazole. These alcohols were obtained by condensing 1,1,3,3-tetraethoxypropane and acetylacetone, respectively, with β-hydroxyethyl hydrazine [36]. Later, 1-vinylpyrazoles were prepared starting from pyrazoles with free NH and different substituents at C-4 by reaction with boiling vinyl acetate in the presence of mercuric(II) sulfate as a catalyst for 1–7 h. The catalyst was directly produced in the reaction medium from mercuric(II) acetate and sulfuric acid added dropwise [37]. The 1-vinylpyrazoles were obtained in very good yields (70–86%). Electron-acceptor groups at C-4 of the pyrazole nucleus increased the acidity of the NH group and accelerated the reaction. In 1970, Trofimenko reported the synthesis of 1-vinylpyrazoles and their analogs containing alkyl substituents on the vinyl group by acid-catalyzed cracking of geminal bis(1-pyrazolyl)alkanes **1,** obtained from the reaction of pyrazole with acetals or ketals [38]. At around 200 °C and in the presence of an acid such as *p-*toluenesulfonic acid, the bis(1-pyrazolyl)alkanes **1** containing β-hydrogens underwent fragmentation to 1-vinylpyrazole **2** and pyrazole **3** (Scheme 1).

Other methods for the preparation of 1-vinylpyrazoles include the dehydrohalogenation of 1-(2-haloethyl)pyrazoles with potassium hydroxide in ethanol [39]. However, the treatment of 1-(2-bromoethyl)-5-hydroxy-(3-methyl- and 3-phenyl)pyrazoles under the same reaction conditions afforded the corresponding 2,3-dihydro-(6-methyl- and -6-phenyl)pyrazolo[3,2-*b*]oxazoles. On the other hand, the treatment of 5-benzoyloxy-1-(2-bromoethyl)-(3-methyl- and -3-phenyl)pyrazoles with sodium *t*-butoxide in butanol gave 5-hydroxy-(3-methyl- and -3-phenyl)-1-vinylpyrazoles. 

**Scheme 2:**
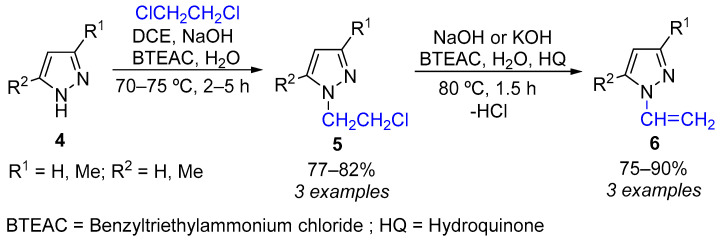
Synthesis of 1-vinylpyrazoles **6** by dehydrochlorination of 1-(2-chloroethyl)pyrazoles **5** [40].

Another interesting method that allowed the formation of 1-vinylpyrazoles used water as solvent under phase transfer catalysis conditions (PTC). This method involved the *N*-alkylation of pyrazole **4** with dichloroethane (DCE) followed by dehydrochlorination of the obtained 1-(2-chloroethyl)pyrazoles **5**, which proceeded smoothly in water under PTC conditions to the formation of 1-vinylpyrazoles **6** in 75–90% yield (Scheme 2) [40]. 

**Scheme 3:**
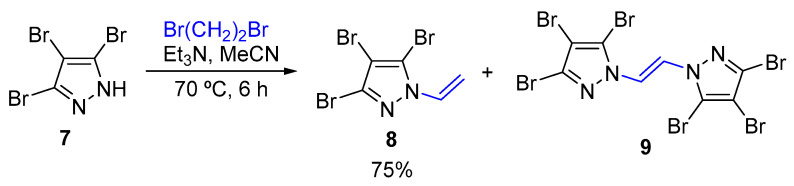
Synthesis of 1-vinylpyrazoles **8** from 3,4,5-tribromopyrazoles **7** and 1,2-dibromoethane [41].

The reaction of 3,4,5-tribromopyrazole **7** with 1,2-dibromoethane and triethylamine followed by the elimination of HBr gave 3,4,5-tribromo-1-vinylpyrazole **8** in 75% overall yield (Scheme 3) [41]. The formation of 1,2-bis(3,4,5-tribromopyrazol-1-yl)ethane **9** by substitution of both bromine atoms of 1,2-dibromoethane could be suppressed by performing the reaction in acetonitrile using a large excess of triethylamine.

**Scheme 4:**
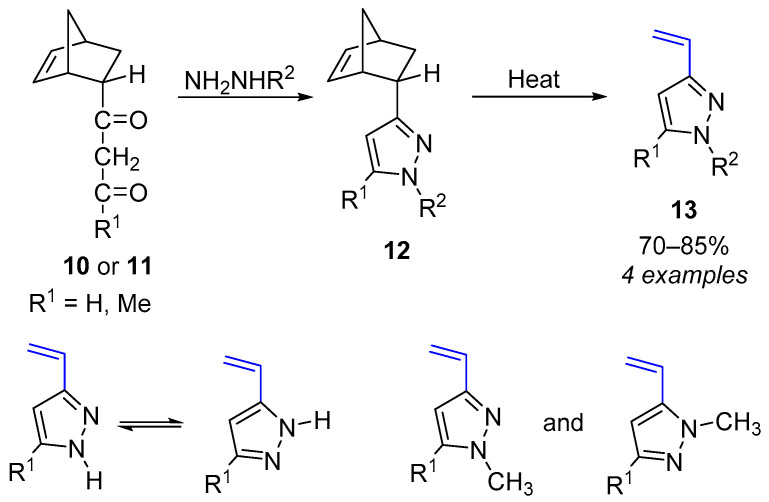
Synthesis of 3(5)-vinylpyrazoles **13** [43].

Only a few examples of the synthesis of 3- and 5-vinylpyrazoles are known, especially when one of the nitrogens bears a proton. In 1976, Sharp reported the synthesis of 3-vinylpyrazoles by thermolysis and rearrangement of 3*H*-1,2-diazepines [42]. Later, Ponticello demonstrated that the cracking of the adducts **12**, prepared by a condensation–cyclization reaction of a β-ketoaldehyde **10** (R^1^ = H) or a β-diketone **11** (R^1^ = Me) with hydrazine or its derivatives, afforded 3(5)-vinylpyrazoles **13** in good yield (Scheme 4) [43]. When R^1^ = H, the two tautomers corresponding to the 3- and 5-vinylpyrazoles are indistinguishable, since very easy interconversion might be expected through ions formed by addition and loss of a proton. As expected, condensation of methyl hydrazine with β-ketoaldehyde or β-diketone each gave two isomeric pyrazoles. Substitution on nitrogen prevents tautomerism; thus, the two isomers were not identical.

**Scheme 5:**
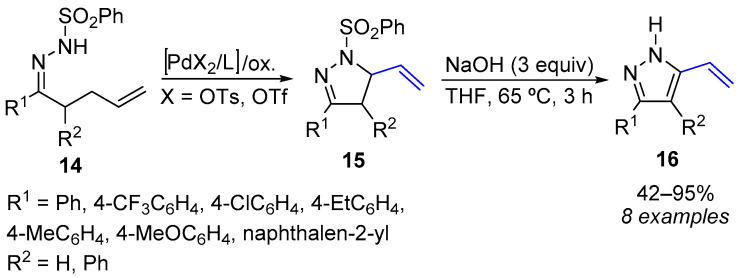
Synthesis of 5-vinylpyrazoles **16** by base-induced eliminative aromatization of 5-vinylpyrazolines **15** [44].

Furthermore, 5-vinylpyrazoles **16** could be obtained by aromatization of 5-vinylpyrazolines **15** generated from *N*-sulfonyl,*C*-homoallyl-hydrazones **14** via Pd-catalyzed C-H oxidative C,N-cyclization through a 5-*exo-trig* process from a π–allyl complex intermediate when the Pd(II) center is associated to noncoordinating anions such as tosylates or triflates. Indeed, 5-vinylpyrazolines with electron-active substituents at the *p*-position of the phenyl ring and hindered pyrazolines could be converted to the corresponding pyrazoles in moderate-to-high yields through a base-induced eliminative aromatization process (Scheme 5) [44].

**Scheme 6:**
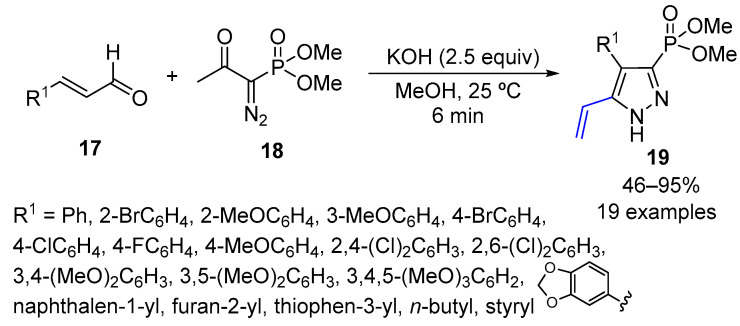
Domino reaction for the synthesis of functionalized 5(3)-vinylpyrazoles **19** using Bestmann–Ohira reagent **18** [45].

Mohanan and coworkers developed a rapid synthesis of 5(3)-vinylpyrazoles under mild conditions. The reaction was based on the versatility and dual reactivity of Bestmann–Ohira reagent (BOR) **18** as a homologation reagent and cycloaddition reactant in a domino reaction with a cinnamaldehyde **17** (Scheme 6) [45]. The sequence involved a formal 1,3-dipolar cycloaddition/Horner–Wadsworth–Emmons (HWE) homologation of the resulting pyrazoline carbaldehydes followed by a 1,3-H shift to give the 5(3)-vinylpyrazoles.

**Scheme 7:**
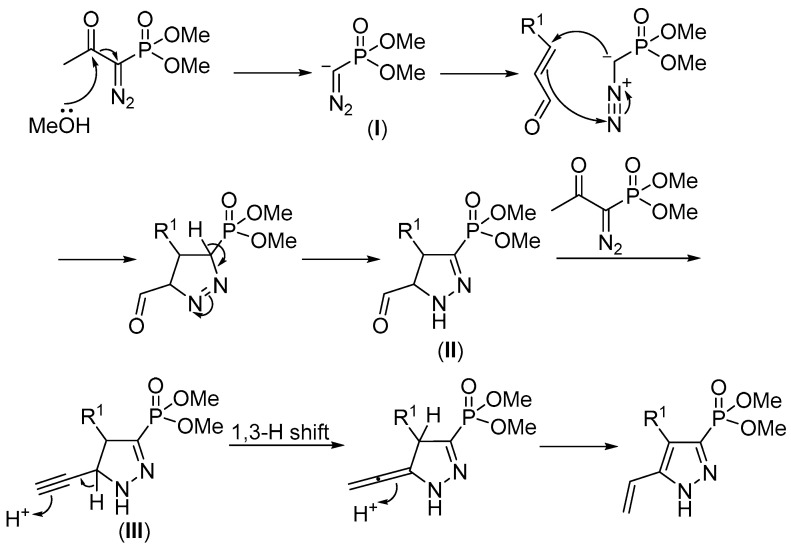
Plausible domino reaction mechanism [45].

The reaction mechanism started with the methanolysis of BOR and generation of a diazomethyl anion **I**. Then, a 1,3-dipolar cycloaddition of **I** to cinnamaldehydes gave pyrazoline carbaldehydes **II**. Reaction of pyrazolines **II** with another molecule of BOR generated pyrazoline alkyne intermediates **III**, which after a 1,3-H shift followed by aromatization produced 5(3)-vinylpyrazoles **19** (Scheme 7).

**Scheme 8:**
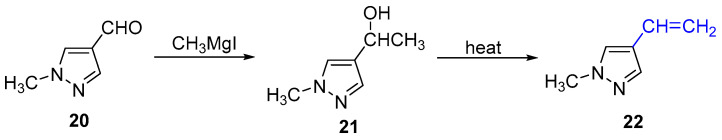
Synthesis of 1-methyl-4-vinylpyrazole **22** from 1-methylpyrazole-4-carbaldehyde **20** [48].

There have also been some reports on the synthesis of 4-vinylpyrazoles. For example, the decarboxylation of β-(1-phenyl-4-pyrazolyl)acrylic acid afforded 1-phenyl-4-vinylpyrazole [46,47]. Another method involved the reaction of 1-methyl-1*H*-pyrazole-4-carbaldehyde **20** with the Grignard reagent methylmagnesium iodide to produce the corresponding alcohol **21**, which upon heating afforded 1-methyl-4-vinyl-1*H*-pyrazole **22** (Scheme 8) [48].

**Scheme 9:**
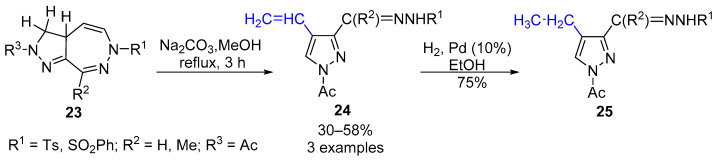
Synthesis of 4-vinylpyrazoles **24** from tetrahydropyrazolo[3,4-*d*][1,2]diazepines **23** [49].

When tetrahydropyrazolo[3,4-*d*][1,2]diazepines (**23**) bearing arylsulphonyl groups at N-6 and acetyl groups at N-2 were treated with methanolic sodium carbonate (1 g per g of **23**), the corresponding 4-vinylpyrazole-3(5)-carbaldehyde tosyl and phenylsulphonylhydrazones **24** were obtained. Catalytic hydrogenation of the vinyl function gave 4-ethylpyrazoles **25** (Scheme 9) [49].

## 3. Reactivity of Vinylpyrazoles

Vinylpyrazole and substituted vinylpyrazoles do not resemble enamines in reactivity. This is in accord with the nonavailability of electrons from the N-1 to stabilize dipolar structures of transition states such as those commonly invoked to account for the reactivity of enamines. Vinylpyrazoles coexist with pyrazole and show no tendency to add it back. The shelf life of vinylpyrazoles is good, and no polymerization in the neat liquid is observed even after 2 years [27,28,29,30,31,32,33,34,35]. Some reactions with vinylpyrazoles have been described in the literature. However, the reactivity of these scaffolds has been barely explored. Some examples of vinylpyrazoles’ transformations are described in the following subsections.

### 3.1. Cycloaddition Reactions

**Scheme 10:**
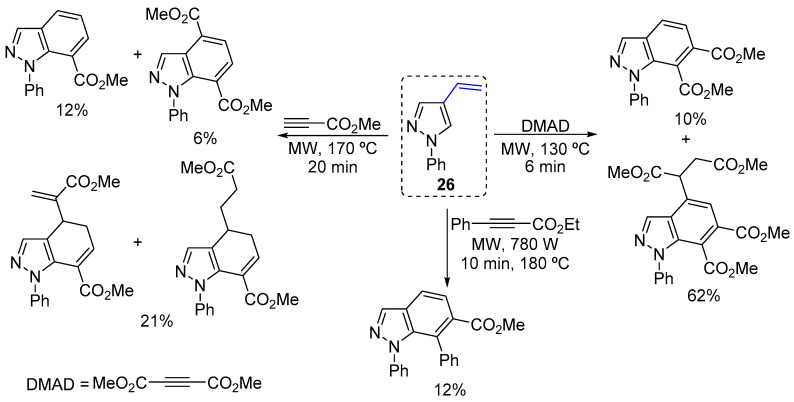
Diels–Alder reactions of 1-phenyl-4-vinylpyrazole **26** with different dienophiles [53].

**Scheme 11:**
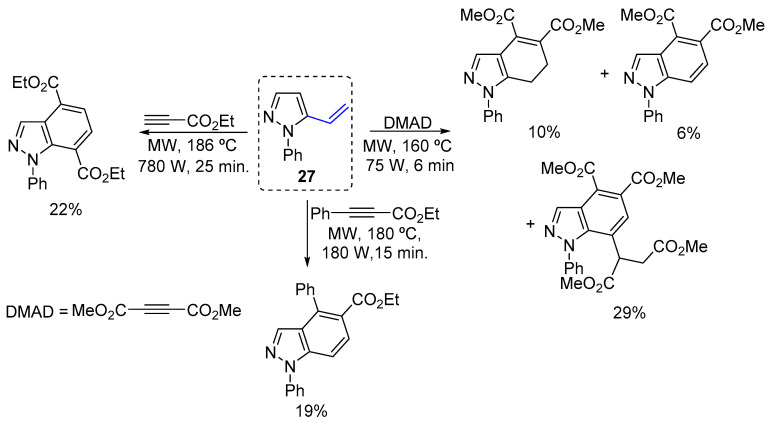
Diels–Alder reactions of 1-phenyl-5-vinylpyrazole **27** with different dienophiles [53].

Diels–Alder (DA) cycloadditions are amongst the most elegant reactions for rapidly building complex cyclic compounds. Vinylpyrazoles are very reluctant to react as dienes in DA cycloadditions because of the loss of aromaticity of the pyrazole ring on [4 + 2] cycloadduct formation being much less reactive than vinylpyrroles and vinylindoles. Harsh reaction conditions are required, such as sealed vessels and high pressures (8–10 atm) and temperatures (120–140 °C) for long reaction times (several days), to obtain very low or moderate yields [50,51]. So far, the vinylpyrazoles have been used as dienes in Diels–Alder reactions for the preparation of compounds with medicinal interest, being these ones of the most described reactions of vinylpyrazoles in literature. In 1996, Díaz-Ortiz et al. studied the effect of microwaves in solvent-free conditions in the improvement of the reactivity of vinylpyrazoles. They found that 4-vinylpyrazoles **26** reacted with dienophiles, such as methyl and ethyl propiolate, dimethyl acetylenedicarboxylate, *N*-phenylmaleimide, or tetracyanoethene, to form 1:1 adducts [52]. The cycloaddition occurred rapidly (6–30 min), and the yields, while generally fair, were superior to those of conventional methods. Moreover, with microwave activation, it was possible to use low-reactive dienophiles such as ethyl phenylpropiolate, and other intermediate products not observed in conventional methods were isolated and characterized. This type of reaction also occurred with 3- and 5-vinylpyrazoles [53]. Since DA reactions of vinylpyrazoles have been the subject of several publications, only some examples are provided for the reaction of 4- and 5-vinylpyrazoles **26** and **27** with some common dienophiles (Scheme 10 and Scheme 11) [53].

Sepúlveda-Arques et al. investigated the effects of the presence of substituents either on the pyrazole ring nitrogen or in the vinyl group on the improvement of the yields of the cycloadducts obtained from the Diels–Alder reactions. They showed that the reactivity was not limited to one kind of substituent on the nitrogen ring but was nonetheless limited and that it failed with small changes by substitution on the vinyl group [54]. 

**Scheme 12:**
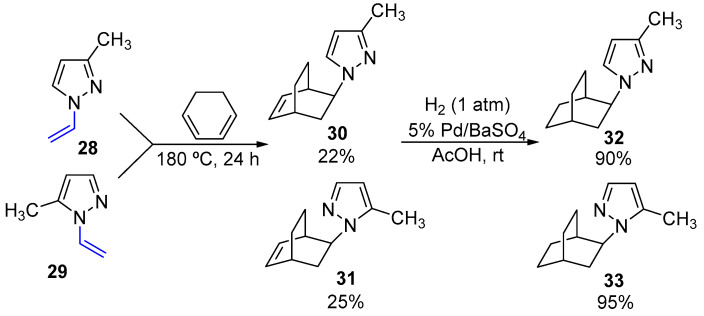
Diels–Alder reaction of 3- and 5-methyl-1-vinylpyrazoles **28** and **29** with cyclohexa-1,3-diene [55].

Few data on the reactivity of 3- and 5-methyl-1-vinylpyrazoles **28** and **29** as dienophiles are available in the literature. Asratyan and coworkers studied the behavior of these pyrazoles as dienophiles in the reaction with cyclohexa-1,3-diene (Scheme 12) [55]. Although ^1^H- and ^13^C-NMR data showed that 5-methyl-1-vinylpyrazole **29** existed mainly as the S-*cis*-N^2^ isomer, while 3-methyl-1-vinylpyrazole **28** was a roughly equimolar mixture of steric isomers [28], no characteristic differences were found in the behavior of these compounds in the cycloaddition. The reaction with the diene proceeded only at 180 °C to give the product in low yields. When reaction temperature is increased to 220 °C, fast polymerization of the diene and dienophile occurred. The authors suggested that the spatial location of the vinyl group in 1-vinylpyrazoles **28** and **29** with respect to N-2 took no part in the Diels–Alder reaction. The obtained products **30** and **31** went through a hydrogenation reaction to afford compounds **32** and **33**.

**Scheme 13:**
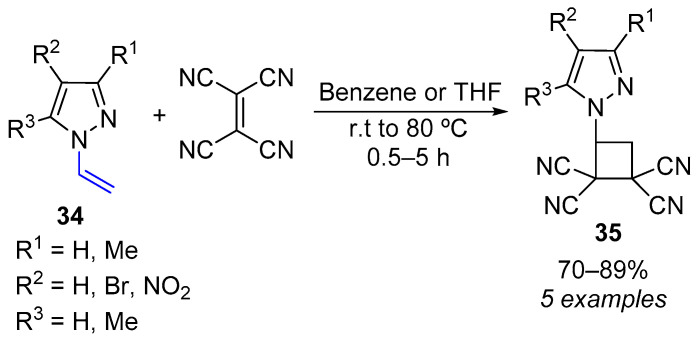
[2 + 2] Cycloaddition of 1-vinylpyrazoles **34** with tetracyanoethylene [56].

In aprotic solvents, 1-vinylpyrazoles **34** reacted with tetracyanoethylene. The reaction gave mainly l-(2,2,3,3-tetracyano-l-cyclobutyl)pyrazoles **35** as a result of a [2 + 2] cycloaddition involving the formation of a π–π complex at the first stage (Scheme 13) [56]. The reaction occurred in benzene at room temperature for 1-vinylpyrazole but required heating at 80 °C for its 3-methyl and 5-methyl derivatives, while 2–5% of the product was obtained for 4-bromo-1-vinylpyrazole, and 3,5-dimethyl-4-nitropyrazole did not react to give the [2 + 2]-cycloaddition product. Using a more polar solvent such as THF or without a solvent, excess vinylpyrazoles gave high yields of the corresponding l-(2,2,3,3-tetracyano-l-cyclobutyl)pyrazoles **35** at room temperature. 

### 3.2. Polymerization Reactions 

Polymerization of vinylpyrazoles has been performed with azo initiators [38]. The rate and extent of polymerization depends on the nature of the substituents on the vinyl group. For example, neat 1-vinylpyrazole polymerizes almost explosively, while 1-(propen-2-yl)pyrazole polymerizes to a lesser extent, and the more heavily substituted the analogs are, the slower the polymerization is. In dilute benzene, 1-vinylpyrazole solution was cleanly polymerized to polymers of molecular weight 150.000–330.000. Furthermore, 1-vinylpyrazole polymerized under free-radical initiation to a high polymer. In this case, the same trend was observed; the extent of polymerization diminished with increasing substitution on the vinyl group. Nikitenko and coworkers studied the free-radical polymerization of 3-methyl-1-vinylpyrazole and 5-methyl-1-vinylpyrazole separately, as individual substances and not as a mixture of isomers [57]. In both cases, the rate of polymerization was proportional to 0.5 order with respect to the initiator azobisisobutyronitrile (AIBN) concentration. When the concentration of monomer was low (≤3M), the reaction followed first-order kinetics, but for higher initial concentrations, the order was lower than the unit. The overall rate of polymerization was found to be higher for 5-methyl-1-vinylpyrazole. 

### 3.3. Halogenation and Hydrohalogenation Reactions

**Scheme 14:**
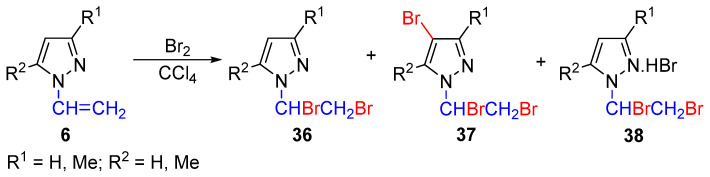
Halogenation and hydrohalogenation of 1-vinylpyrazoles **6** [58].

Compared with 1-vinylimidazoles and 1-vinyltriazoles, 1-vinylpyrazoles show a different behavior in bromination reactions [58]. With 1-vinylimidazoles, the formation of a complex with the halogen occurs, while with 1-vinyltriazoles, addition of bromine to the double bond of the vinyl group occurs. Notably, 1-vinylpyrazoles **6** do not form complexes with bromine. The bromination in carbon tetrachloride at −20 °C affords a complex mixture of products that is difficult to separate. The main reaction products are 1-(1’,2’-dibromo)ethylpyrazoles **36** and the product of electrophilic substitution at C-4 **37**; however, the coordination of the released hydrogen bromide with the pyrazoles present in the reaction mixture also produces hydrohalides **38** (Scheme 14). Higher percentages of **36** can be obtained by increasing the temperature, by adding the vinylpyrazole to a solution of bromine, and by using an excess of bromine, but the stability of the bromination products depends on the position and number of substituents in the pyrazole ring, especially methyl groups.

**Scheme 15:**
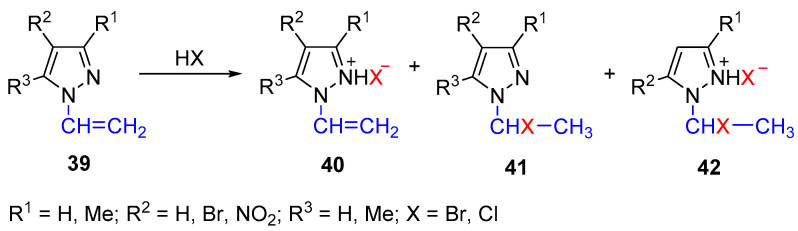
Main products formed in the hydrohalogenation of 1-vinylpyrazoles **39** [59].

The products of the hydrohalogenation of vinylpyrazoles depend on the structure of the pyrazole itself and the nature of the hydrogen halide. Skvortsova and coworkers investigated the hydrohalogenation of a series of vinylpyrazoles **39**, (l-vinylpyrazole, 4-bromo-l-vinylpyrazole, 3-methyl-l-vinylpyrazole, 5-methyl-l-vinylpyrazole, 3,5-dimethyl-l-vinylpyrazole, and 4-nitro-3,5-dimethyl-l-vinylpyrazole) (Scheme 15) [59]. At −150 °C, addition of hydrogen halides to all l-vinylpyrazoles occurred only at the N-2 with formation of salts **40**. However, the hydrohalides of the less basic vinylpyrazoles (l-vinylpyrazole, 4-bromo-l-vinylpyrazole, and 4-nitro-3,5-dimethyl-l-vinylpyrazole) decomposed rapidly. On the other hand, at 20–25 °C, l-vinylpyrazole and 4-bromo-l-vinylpyrazole added predominantly hydrogen halides at the double bond of the vinyl group, with formation of l-(l′-haloethyl)pyrazoles **41**, which was in accordance with Markovnikov’s rule. Then, the resulting compounds **41** underwent further hydrohalogenation at the N-2 to give compounds **42**. Under these conditions, the more basic alkyl-substituted l-vinylpyrazoles produced the corresponding hydrohalides. Addition of hydrogen halides to the double bond of the vinyl group of 4-nitro-3,5-dimethyl-l-vinylpyrazole was more difficult, since the nitro group decreased the nucleophilicity not only of the N-2 but of the double bond of the vinyl group. The reaction of 4-bromo-l-vinylpyrazole with HBr formed a mixture of products, whereas the reaction with HCl proceeded most specifically [59].

### 3.4. Difluorocyclopropanation Reactions

**Scheme 16:**
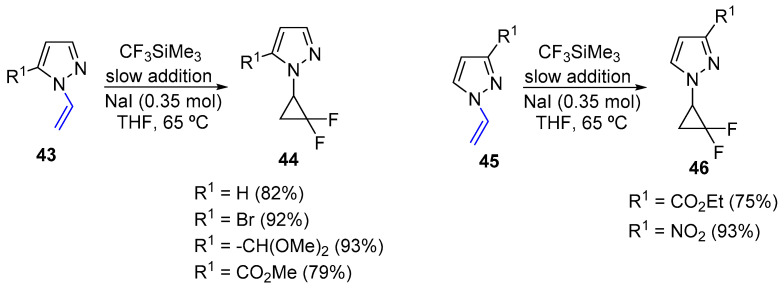
Difluorocyclopropanation of 1-vinylpyrazoles **43** and **45** [64].

Fluorinated cyclopropanes have become extraordinary structural motifs with huge importance in organic synthesis, drug discovery, and agrochemistry. In fact, highly potent compounds bearing a *gem*-difluorocyclopropyl group were disclosed in recent patents [60,61,62,63]. In most of the compounds reported in the literature, the *gem*-difluorocyclopropyl is attached to a carbon atom, while the corresponding *N*-linked analogues are less common. *N*-vinylpyrazoles **43** and **45** undergo difluorocyclopropanation with CF_3_SiMe_3_-NaI system with formation of the corresponding *N*-difluorocyclopropyl derivatives **44** and **46** in very good yield (Scheme 16). These compounds are stable and undergo further regioselective functionalization to afford *gem*-difluorocyclopropylpyrazole amines, carboxylic acids, aldehydes, bromides, and boronic derivatives [64]. It is noteworthy that this method is not successful for the preparation of other azole derivatives, such as imidazoles, triazoles, and tetrazoles, possibly because of the presence of a nucleophilic nitrogen atom (in the case of the imidazole) or electronic effects for the other representatives.

### 3.5. Phosphorylation Reactions

**Scheme 17:**
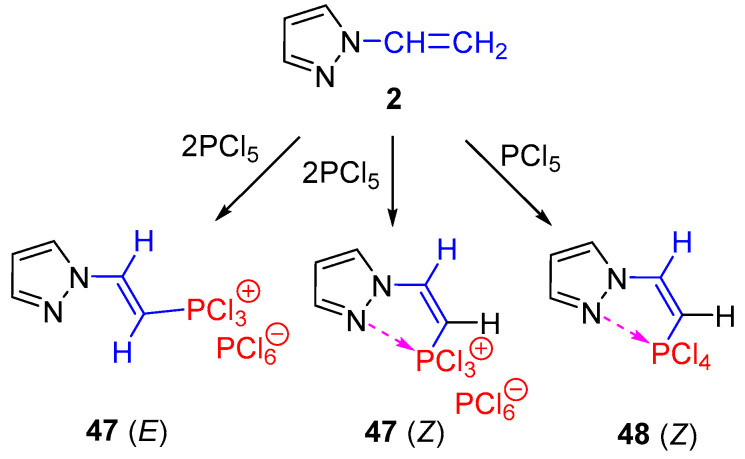
Phosphorylation of 1-vinylpyrazole **2** [65].

The phosphorylation of 1-vinylpyrazole **2** with phosphorus pentachloride afforded phosphorylated azoles **47** and **48** (Scheme 17) [65]. Krivdin and coworkers studied the effects of intramolecular and intermolecular coordination on ^31^P nuclear shielding both experimentally and theoretically, with a special emphasis on the calculation of ^31^P shielding constants. They noticed dramatic ^31^P nuclear shielding to approximately 150 ppm on changing the phosphorus coordination number by one (+(90–120) ppm for tetracoordinated phosphorus, −(20–40) ppm for pentacoordinated phosphorus, and −(200–220) ppm for hexacoordinated phosphorus) and suggested that ^31^P-NMR chemical shifts could serve as an unambiguous criterion of intra- or intermolecular coordination involving phosphorus.

### 3.6. Ring-Closing Metathesis Reactions

**Scheme 18:**
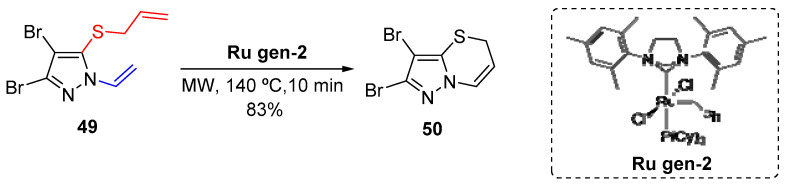
Ring closure metathesis reaction of 1-vinylpyrazole **49** with formation of 5*H*-pyrazolo[5,1-*b*][1,3]thiazine **50** [41].

The *N*-vinylated pyrazoles are also useful intermediates for Grubbs’ ring closure metathesis reaction (RCM) to generate novel heterocycles [41,66]. For example, 5*H*-pyrazolo[5,1-*b*][1,3]thiazine **50** was obtained in 83% yield, in a short reaction time, by microwave irradiation of 1-vinylpyrazole **49** with Grubbs’ second-generation catalyst (**Ru gen-2**) (Scheme 18) [41,67], which in this reaction was more reactive than Grubbs’ first generation [68,69] and the Hoveyda–Grubbs’ catalyst [70].

### 3.7. Organomettalic Reactions

**Scheme 19:**
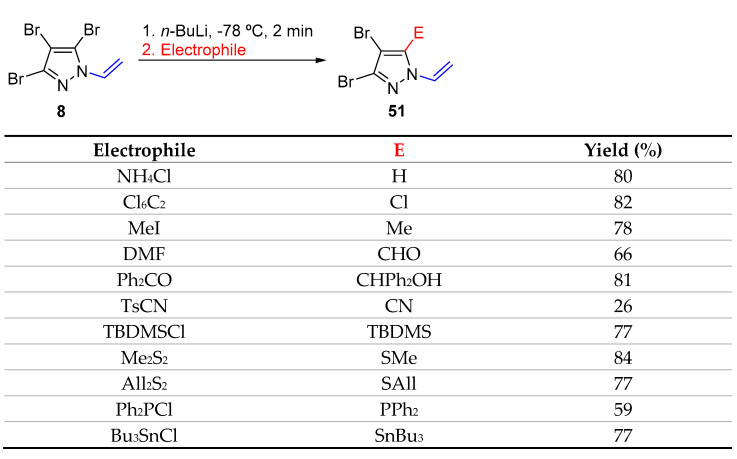
Bromine–lithium exchange of 3,4,5-tribromo-1-vinylpyrazole **8** and reactions with different electrophiles [41].

The vinyl group is a stable and versatile *N-*protection group for bromine–lithium exchange reactions in pyrazoles that can be removed easily under mild conditions. Begtrup and coworkers demonstrated that 3,4,5-tribromo-1-vinylpyrazole **8**, underwent regioselective bromine–lithium exchange at the 5-position. Subsequent addition of an electrophile gave 5-substituted 3,4-dibromo-1-vinylpyrazoles **51** (Scheme 19) [41]. A range of electrophiles can be introduced at the 5-position. Longer lithiation time (10–15 min) led to lower yields of the 5-substituted product because of the low stability of the pyrazole anion.

**Scheme 20:**
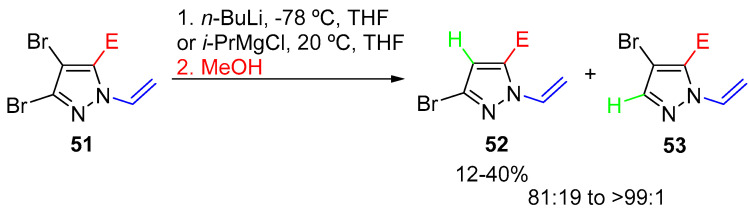
Regioselectivity of bromine–lithium and bromine–magnesium exchange in 5-substituted 3,4-dibromo-1-vinylpyrazoles **51** [41].

The 5-substituted 3,4-dibromo-1-vinylpyrazoles **51** can undergo subsequent bromine–lithium or bromine–magnesium exchange using *n*-BuLi or *i-*PrMgCl together with protons from methanol as the electrophile, affording compounds **52** and **53**. The reaction occurred preferentially at C-4, with the regioselectivity between C-3- and C-4 being influenced by the nature of the metal and the 5-substituent (Scheme 20) [41]. Begtrup and coworkers found that bromine–lithium exchange took place with higher regioselectivity than bromine–magnesium exchange.

**Scheme 21:**
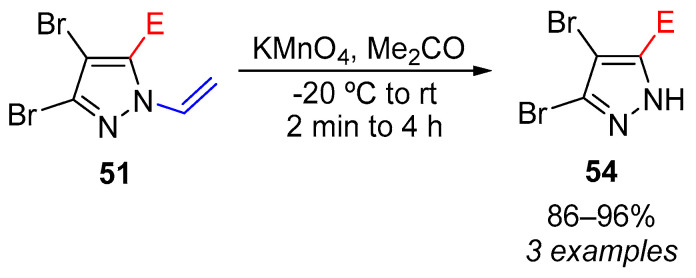
Cleavage of the vinyl group of 5-substituted 1-vinylpyrazoles **51** [41].

The vinyl group can be removed from the 5-substituted compounds **51** by mild treatment with KMnO_4_, affording the corresponding NH-pyrazoles **54**. Depending on the substituents, slightly different conditions may be required (Scheme 21) [41]. For example, the vinyl group of 3,4,5-tribromo-1-vinylpyrazole (E = Br) and 3,4-dibromo-5-methyl-1-vinylpyrazole (E = Me) could be removed smoothly in excellent yield (96% for both) by treatment with a 2% solution of KMnO_4_ at room temperature and at 10 °C. When oxidation sensitive groups such as SMe (E = SMe) are present, the devinylation should be performed at −20 to −10 °C to avoid concurrent oxidation of SMe to SO_2_Me.

### 3.8. Transition-Metal-Catalyzed Reactions

Transition-metal-catalyzed reactions have gained increasing interest in organic chemistry over the last years because of their versatility and high levels of chemo-, regio-, and stereoselectivity. Many transition-metal complexes of Pd, Au, Zn, Co, Rh, Ru, Mo, Ni, Cu, and Fe have been developed as catalysts to accelerate these organic reactions. One of the hottest topics in transition metal catalysis is the development of highly efficient catalysts for direct C-H bond functionalization reactions. Preferentially, these reactions should be performed in mild conditions (room temperature, weak base, air atmosphere) to allow easy access to nitrogen-containing compounds such as pyrazoles frequently found in pharmaceuticals, crop-protection chemicals, and products for material sciences.

#### C-H Activation Reactions

**Scheme 22:**
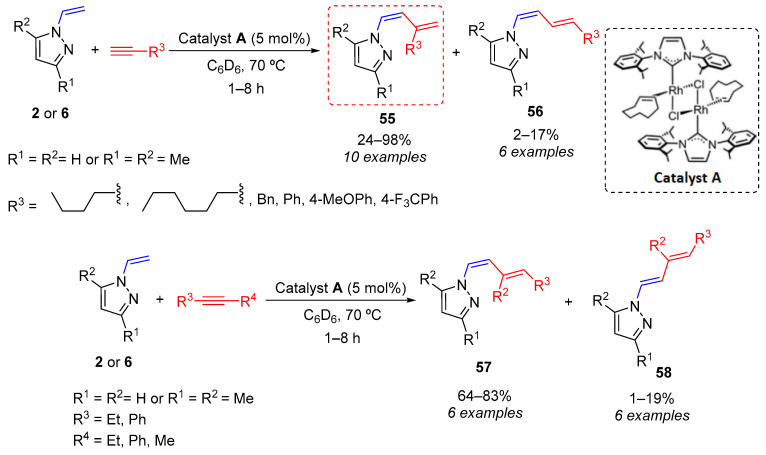
Coupling reaction of 1-vinylpyrazoles **2** and **6** with terminal and internal alkynes mediated by Rh-NHC catalyst [73].

**Scheme 23:**
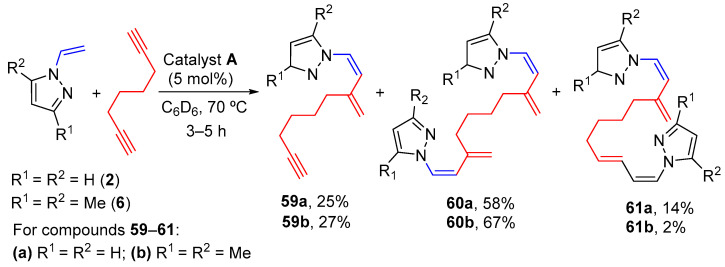
Coupling reaction of 1-vinylpyrazoles **2** and **6** with terminal diynes mediated by Rh-NHC catalyst [73].

Among the transition-metal-catalyzed reactions, C-C bond formation via C-H activation has gaining increasing interest as a very powerful, selective, and atom-economical tool in organic synthesis [71]. Aromatic protons have been commonly involved in this process, but functionalization of the vinyl C-H bond is more challenging because of competitive polymerization or Michael addition reactions, and electron-rich olefins are much less reactive [72]. The 1-vinylpyrazoles **2** and **6** underwent coupling with alkynes via C-H activation to afford Markovnikov-selective butadienylpyrazole derivatives **55** and **57** under mild conditions [73]. This reaction was efficiently catalyzed by the rhodium(I)-*N*-heterocyclic carbene catalyst **A** (A = [Rh(*μ*-Cl) (*I*Pr) (*η*^2^-coe)]_2_) (Rh-NHC). The reaction occurred both with terminal or internal alkynes (Scheme 22) and with terminal diynes (Scheme 23) in mild conditions. With terminal diynes, a mixture of monohydrovinylated *Z*/*gem* product **59** and the doubly coupled derivatives bis-*Z*/*gem* **60** and *Z*/*gem Z*/*E* **61** was obtained (Scheme 23). The presence of the carbene ligand in the rhodium catalyst was found to be essential for the catalytic coupling and C-H activation of the electron-rich pyrazolyl olefin. Moreover, 1-vinylpyrazole **2** (R^1^ = R^2^ = H) was more reactive than 3,5-dimethyl-1-vinylpyrazole **6** (R^1^ = R^2^ = Me), but the reaction with the former presented slightly lower selectivity. It is worth mentioning that even with the diynes, the selectivity trend towards Markovnikov addition products was maintained, with disubstituted pyrazole **6** being slightly more selective than **2**. Contrarily to aromatic alkynes, aliphatic terminal alkynes were preferentially hydrovinylated without dimerization, cyclotrimerization, or polymerization of the alkyne.

**Scheme 24:**
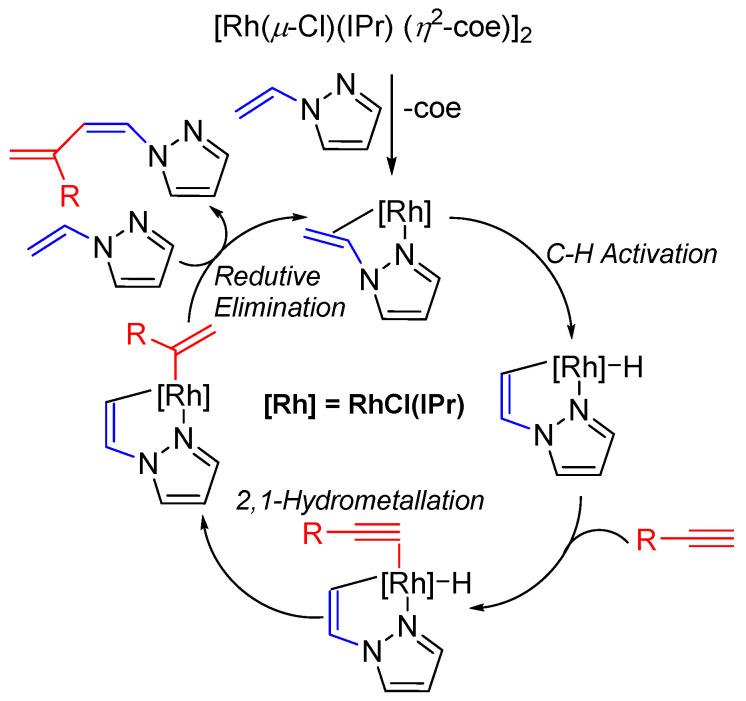
Plausible catalytic cycle for the coupling reaction of 1-vinylpyrazole **2** with alkynes [73].

The reaction mechanism started with the C-H activation of the vinylpyrazole assisted by nitrogen coordination to the metallic center. Then, alkyne coordination, insertion, and reductive elimination took place to afford the coupling product (Scheme 24) [73].

### 3.9. Miscellaneous

#### 3.9.1. Reaction with Ethyl *N*-Trichloroethylidenecarbamate

**Scheme 25:**
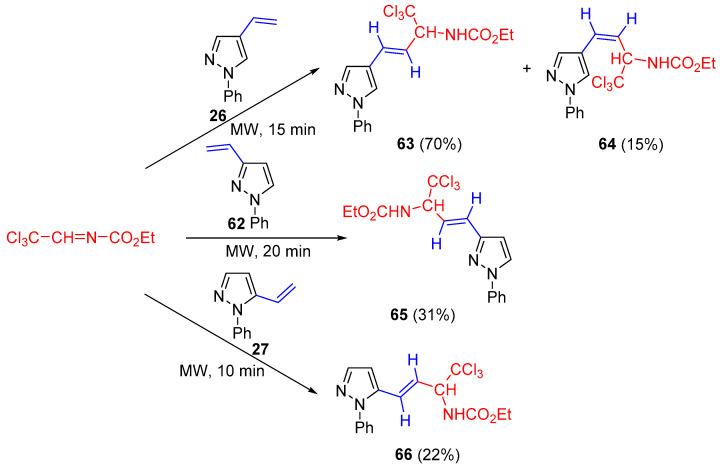
Reactions of 1-phenylvinylpyrazoles **26**, **62**, and **27** with ethyl *N*-trichloroethylidenecarbamate [74].

The reaction of vinylpyrazoles **26**, **62**, and **27** with *N*-trichloroethylidenecarbamate, which participates in cycloaddition reactions as a dienophile, as a dipolarophile, and even as a heterodiene, did not afford any cycloaddition product. Under microwave heating, the result of the reaction was found to depend on the nature of the diene and the substitution of the pyrazole ring. In fact, an electrophilic substitution reaction occurred through the exocyclic double bond, which was activated by conjugation with the pyrazole ring, to give Michael addition to the conjugated imine system (Scheme 25) [74]. Even in the 3- and 5-substituted pyrazoles **62** and **27**, the reaction occurred at the double bond and not in the activated C-4 of the pyrazole ring. A mixture of *trans-***63** and *cis-***64** isomers was otained with pyrazole **26** (although the *cis* was obtained in 15% yield), while the thermodynamic *trans* isomer **65** and **66** was the only product observed in the reactions of pyrazoles **62** and **27,** respectively. Under conventional heating in an oil bath, no reaction occurred under similar conditions of temperature and time, and the starting vinylpyrazoles were not recovered because of dimerization in these conditions.

#### 3.9.2. Reaction with Alkanethiols

Pyrazoles containing sulfur atoms have interesting biological activities and are used as drugs [75]. This type of pyrazoles can be prepared from vinylpyrazoles and their alkyl derivatives, which are known to react with thiols via either ionic or free radical mechanisms, with the formation of α- and β-addition products, depending on whether the addition follows the Markovnikov rule or not, respectively [76].

The method of radical thiylation yields more stable products and is easily and rapidly conducted not only with heating, catalysts, and irradiation with UV light but at 20 °C without special initiation, being a more convenient method of synthesis of pyrazoles with sulfur-containing substituents.

**Scheme 26:**
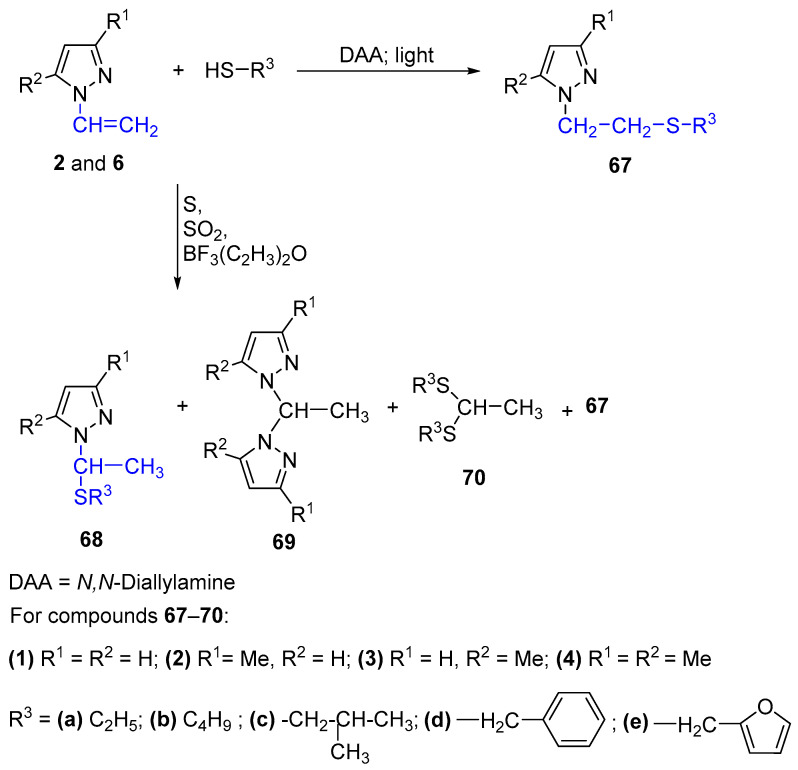
Reactions of 1-vinylpyrazoles **2** and **6** with alkanethiols [76].

β-Addition products, the 1-(pyrazolyl-1)-2-(alkylthio)ethanes (Scheme 26, **67.1a**–**e**–**67.3a**–**e**, **67.4b,d,e**), were formed, in 80–85% yield, in the reaction of thiols with 1-vinylpyrazoles **2** and **6**. The ease of the radical addition was a function of both the reactants and the reaction conditions, namely the temperature. Methyl substituted 1-vinylpyrazoles **6** reacted more energetically than **2**, and the reaction time was reduced to 0.5–2 h by increasing the temperature to 80 °C and using AIBN (1%) as a radical initiator. In the presence of ionic initiators (BF_3_(C_2_H_5_)_2_O, SO_2_, S) with heating, the reaction followed two competing pathways, with the formation of a mixture of α- and β-addition products **68** and **67**, the ratio of which depended on the reaction conditions used, together with 1,1-bis(pyrazol-1-yl)ethanes **69** and 1,1-bis(*R*-thio)ethanes **70**, formed as a result of disproportionation of 1-(pyrazol-1-yl)-1-(*R*-thio)ethanes **68.1a**–**d**, **68.4a**–**d**. The authors isolated some products of α-addition **68.2b**–**68.4b**, **68**.**4a,c,d**, thioacetals **70a**–**c**, and pyrazoles **69.1**–**69.4**. Thiylation of vinylpyrazole **6.4** (R^1^ = R^2^ = Me) with butane-1-thiol (**b**) afforded only the product of β-addition **67.4b**, with *p-*toluenesulfonic acid (3%, 9%, 90 °C, 8 h). In the presence of elemental sulfur, the addition of thiols to alkenes followed the Markovnikov rule, and sulfur inhibited radical processes. Total yields of thiylation for vinylpyrazole **67.4b** were no greater than 53% in the presence of 3, 9, and 15 mol% of sulfur in heating at 90 °C for 14 h. Increasing the temperature to 120 °C, the yield increased to 80–83%. In similar conditions (120 °C, 14 h, 9 mol%), the yield for the formation of vinylpyrazoles **67**.**1**–**67.3** was slightly lower (70–75%), and the concentration of α- and β-addition products was 95:5–90:10. When BF_3_(C_2_H_5_)_2_O was used (9 mol%, 120 °C, 14 h), the product of the β-addition was the main compound. Addition of radical process inhibitors such as benzoquinone or hydroquinone (3–6%) allowed increasing the concentration of α-products **68** to 95–98%, although β-addition was not completely suppressed.

#### 3.9.3. Reaction with Dichlorocarbene

**Scheme 27:**
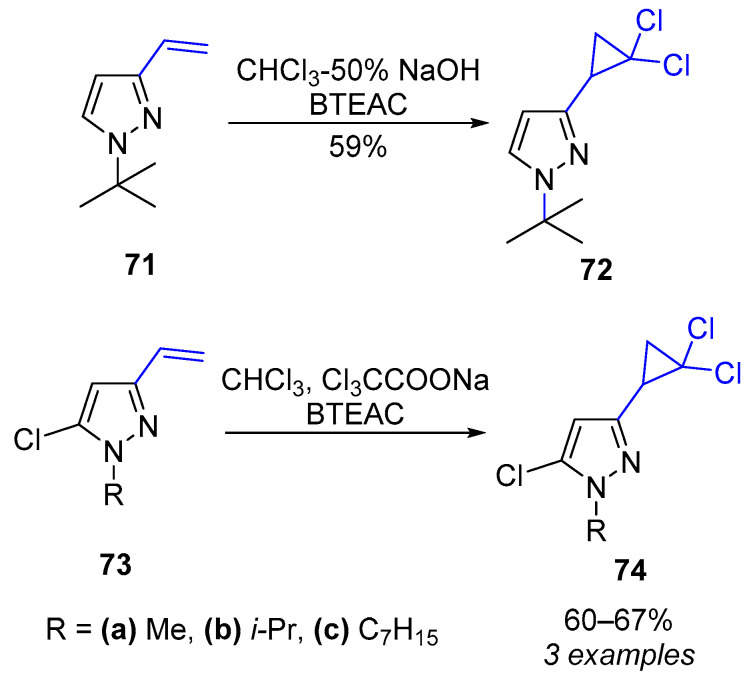
Synthesis of 3-(2,2-dichlorocyclopropyl)pyrazoles **72** and **74** [77].

Furthermore, 3-vinylpyrazoles **71** react with dichlorocarbene, generated from chloroform or sodium trichloroacetate, to afford new cyclopropylpyrazoles **72** [77], which are interesting precursors of bisheterocycles such as 5-(pyrazol-4-yl)isoxazolines. Popov and coworkers synthesized 1*-t*-butyl-3-(2,2-dichlorocyclopropyl)-1*H*-pyrazole **72** by reaction of vinylpyrazole **71** with chloroform and sodium hydroxide under PTC. The reaction was carried out at 60 °C (10 min), and compound **72** was obtained in 59% yield (Scheme 27). The reactions of 1-alkyl-5-chloro-3-vinyl-1*H*-pyrazoles **73a**–**c** under analogous conditions did not afford the target dichlorocyclopropane derivatives. In fact, 1-alkyl-5-chloro-3-(2,2-dichlorocyclopropyl)-1*H*-pyrazoles **74a**–**c** were obtained in good yield only when dichlorocarbene was generated under neutral conditions, by thermal decomposition of sodium trichloroacetate in chloroform in the presence of benzyltriethylammonium chloride (BTEAC) as PTC (Scheme 27) [77].

## 4. Conclusions

Few methods have been reported in the literature for the synthesis of vinylpyrazoles, especially for the preparation of 3(5)- and 4-vinylpyrazoles. In addition, most of the reported methods used harsh conditions and/or toxic reagents and suffered from a lack of generality and/or substrate scope. Thus, novel methods for the synthesis of these interesting pyrazole scaffolds are highly desirable. Furthermore, reactivity studies with vinylpyrazoles have been scarce and mainly restricted to Diels–Alder cycloadditions, polymerization reactions, and halogenation and dehydrohalogenation reactions. The application of the novel tools and concepts of modern organic chemistry to vinylpyrazoles, namely solid supported synthesis, transition-metal catalysis using the more classical and novel catalysts, and visible-light-photoinduced and greener organometallic reactions, may open great opportunities for the development of novel methods of synthesis and transformations of vinylpyrazoles. From our point of view, of high interest will be the investigation of transition-metal-catalyzed reactions of vinylpyrazoles, and especially the more challenging C-H activation reactions, visible-light-photoinduced reactions, and greener organometallic reactions that allow the introduction of different electrophiles in the pyrazole ring, towards the development of more environmentally friendly, atom-economic, and selective transformation of vinylpyrazoles into more advanced molecules with therapeutical interest. 

## Data Availability

Not applicable.
